# Assessing the Relationship between *Helicobacter pylori* and Chronic Kidney Disease

**DOI:** 10.3390/healthcare9020162

**Published:** 2021-02-03

**Authors:** Koichi Hata, Teruhide Koyama, Etsuko Ozaki, Nagato Kuriyama, Shigeto Mizuno, Daisuke Matsui, Isao Watanabe, Ritei Uehara, Yoshiyuki Watanabe

**Affiliations:** 1 Department of Epidemiology for Community Health and Medicine, Kyoto Prefectural University of Medicine, 465 Kajii-cho, Kamigyo-ku, Kyoto 602-8566, Japan; s.caballero1229@gmail.com (K.H.); ozaki@koto.kpu-m.ac.jp (E.O.); nkuriyam@koto.kpu-m.ac.jp (N.K.); d-matsui@koto.kpu-m.ac.jp (D.M.); ricky@koto.kpu-m.ac.jp (I.W.); ruehara@koto.kpu-m.ac.jp (R.U.); watanabe@koto.kpu-m.ac.jp (Y.W.); 2 Endoscopy Department, Kindai University Nara Hospital, 1248-1 Otoda-cho, Ikoma, Nara 630-0293, Japan; s-mizuno@med.kindai.ac.jp

**Keywords:** atrophic gastritis, chronic kidney disease, *Helicobacter pylori*, inter-organ interactions

## Abstract

The relationship between *Helicobacter pylori* infection and/or gastric disorders and chronic kidney disease (CKD) has not been elucidated. We investigated the relationship between *Helicobacter pylori* and/or atrophic gastritis (AG) and chronic kidney disease. In total, 3560 participants (1127 men and 2433 women) were eligible for this cross-sectional study. We divided participants into four study groups: with/without *Helicobacter pylori* infection and with/without AG. The HP (+) AG (−) group demonstrated a significant association with CKD compared with the HP (−) AG (−) group (adjusted odds ratio, 1.443; 95% confidence interval, 1.047–1.989). In contrast, the HP (+) AG (+) group showed significantly lower adjusted odds of CKD than the HP (−) AG (−) group (adjusted odds ratio, 0.608; 95% confidence interval, 0.402–0.920). *H. pylori* infection without AG might be associated with CKD in these participants. Conversely, the HP (+) AG (+) group had lower odds of CKD. Uncovering an association between gastric and renal conditions could lead to development of new treatment strategies.

## 1. Introduction

The stomach is reported to be associated with conditions affecting other organs. Such as, an association between atrophic gastritis (AG) and coronary artery disease has been described [1,2], with AG representing a potential independent risk factor for coronary artery disease [3]. Some studies have reported that *Helicobacter pylori* (*H. pylori*) infection is associated with the metabolic syndrome and abnormal lipid profiles [4,5], while other reports suggest a potential association between *H. pylori* infection and insulin resistance [6,7], liver disorders [8] and ventilator-associated pneumonia [9]. In addition, we have previously suggested that *H. pylori* infection and AG are useful for risk assessment of osteoporosis [10]. These studies suggested that *H. pylori* infection and/or AG affect not only the stomach but also other organs.

A previous study reported that individuals infected with *H. pylori* had a higher risk of subsequent renal dysfunction than those not infected [11]. Conversely, the *H. pylori* infection rate is lower in patients with peptic ulcer disease and concomitant chronic kidney disease (CKD) than in those without CKD [12]. However, the relationship between *H. pylori* infection and/or gastric disorders and CKD has not been elucidated. Therefore, in this study, we investigated the relationship between *H. pylori* and/or AG and CKD, in order to determine whether an association exists between the stomach and kidneys.

## 2. Materials and Methods

### 2.1. Study Population

**Figure 1:**
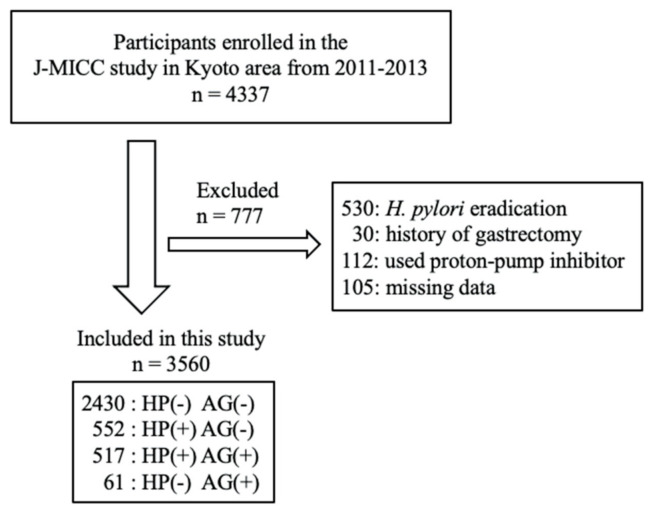
Flow chart of the study participants.

This cross-sectional study included 4337 individuals enrolled in the Japan Multi-Institutional Collaborative Cohort Study in the Kyoto area, from 2011–2013. The Japan Multi-Institutional Collaborative Cohort Study is a new cohort study, launched in 2005, to examine gene–environmental interactions in lifestyle-related diseases [13]. We excluded participants who had received *H. pylori* eradication therapy (*n* = 530) or who had undergone gastrectomy (*n* = 30). Further, participants who used a proton-pump inhibitor (*n* = 112), or who had missing medical information data (*n* = 105), were excluded. This left 3560 participants (1127 men and 2433 women) eligible for analysis. Figure 1 shows flow chart of the study participants. The study protocol was approved by the ethics board of Kyoto Prefectural University of Medicine (ethical approval number, RBMR-E-289), and written informed consent was obtained from all participants.

The study evaluated medical information obtained via self-administered questionnaires. Metabolic equivalents (METs) were assessed, as previously reported [14]. Furthermore, blood chemistry data—triglycerides, total cholesterol, high-density lipoprotein (HDL) cholesterol, low-density lipoprotein (LDL) cholesterol, glucose, haemoglobin A1c (HbA1c), uric acid, blood urea nitrogen, creatinine, and pepsinogen (PG) I/II—along with early morning spot urine samples, collected on the day of survey, were assessed. The estimated glomerular filtration rate (eGFR) was calculated using the following equation (JAP-Creatinine): eGFR (mL/min/1.73 m^2^) = 194 × creatinine^−1.094^ × year^−0.287^ (for men) and eGFR (mL/min/1.73 m^2^) = 194 × creatinine^−1.094^ × year^−0.287^ × 0.739 (for women) [15]. The prevalence of CKD was determined for CKD stages 3–5 (defined as eGFR < 60 mL/min/1.73 m^2^). Anthropometry data was obtained during the clinical examination. Medical history and medication use were assessed by means of a questionnaire. Hypertension was defined as a resting systolic blood pressure ≥ 140 mmHg or if receiving medication, diabetes mellitus (DM) as HbA1c ≥ 6.5% or if receiving medication, dyslipidemia as LDL-cholesterol ≥ 140 mg/dL and HDL-cholesterol < 40 mg/dL or if receiving medication, and anaemia as haemoglobin ≤ 13.0 g/dL in men and ≤ 12.0 g/dL in women.

Participants were divided into four study groups according to a combination of serum anti-*H. pylori* antibody (HP) positivity and levels of serum PG. This method has recently been used in Japan for gastric cancer screening of high-risk individuals [16,17,18]. In brief, blood samples were obtained, and the serum was separated to measure anti-*H. pylori* antibodies (HP) and PG levels. AG was defined according to the serum PG I and II criteria proposed: when a participant fulfilled the criteria of both serum PG I value ≤ 70 ng/mL and PG I/II ratio ≤ 3.0, he or she was diagnosed as having AG. Infection with *H. pylori* was diagnosed using a microplate enzyme immunoassay kit (E Plate Eiken *H. pylori* Antibody, Eiken Chemical, Tokyo, Japan) [19]. Serum samples were analysed according to the manufacturer’s instructions. Participants with a measured value >10 U/mL were considered to be infected with *H. pylori*, i.e., HP (+). The control group consisted of participants who tested HP (−) AG (−); while the other three groups consisted of participants who tested HP (+) AG (−), HP (+) AG (+), or HP (−) AG (+).

### 2.2. Statistical Analysis

Analyses were performed using SPSS statistical software (PASW 25.0). For all analyses, *p* values < 0.05 were considered statistically significant. Continuous variables were expressed as means ± standard deviations (SD), and categorical data were expressed as sums and percentages. Inter-group comparisons were performed using the one-way analysis of variance for continuous variables, or the chi-squared test for categorical variables. Categorical variables included sex, alcohol use, smoking, hypertension, DM, dyslipidemia, stroke, myocardial infarction and/or stenocardia, and anaemia. Odds ratios (ORs) and 95% confidence intervals (CI) were calculated using logistic regression methods in which CKD was the dependent variable and year, sex, body mass index (BMI), METs, smoking, alcohol use, hypertension, DM, dyslipidemia, stroke, myocardial infarction and/or stenocardia, and anaemia were the independent variables.3.

## 3. Results

Table 1 shows the characteristics of participants according to *H. pylori* infection and AG. The mean age of the control group was 50.0 years, vs. > 54.0 years for the other groups. Table 2 shows the distribution of HP, AG, and CKD within age strata. In each group, the prevalence of CKD increased as year increased. However, CKD prevalence in the HP (+) AG (−) group in the age stratum 50–59 years was approximately double that of the control group.

Table 3 shows the proportion of participants with/without CKD, stratified by *H. pylori* infection and diagnosis of AG. Relative to the control group, the HP (+) AG (−) group had an adjusted OR for CKD of 1.465 (95% CI, 1.066–2.012) adjusted for yeae and sex; 1.439 (95 % CI, 1.046–1.979) adjusted for year, sex, BMI, METs, alcohol use, and smoking; and 1.443 (95% CI, 1.047–1.989) adjusted for year, sex, BMI, METs, smoking, alcohol use, hypertension, stroke, dyslipidemia, myocardial infarction and/or stenocardia. There was no significant difference in the adjusted odds of CKD between the control group and the HP (+) AG (−) group—adjusted for lifestyle factors (BMI, METs, alcohol use, and smoking) or medical history (hypertension, stroke, dyslipidemia, myocardial infarction and/or stenocardia). In contrast, we compared the HP (+) AG (+) group and HP (−) AG (−) group, the HP (+) AG (+) group had lower an adjusted OR for CKD of 0.610 (95% CI, 0.406–0.917) adjusted for year and sex. There was no significant difference in the odds of CKD between the control group and the HP (+) AG (+) group, adjusted for lifestyle factors or medical history. On the other hand, the HP (−) AG (+) group showed no significant association with CKD.

## 4. Discussion

Associations between two or multiple organs are essential for the human body to maintain homeostasis and to function normally. Elucidating these associations may be important for clinical decision-making and the development of appropriate treatments for some diseases. With knowledge of interactions between organ systems, we can treat diseases focusing not only on the symptomatic organ, but also on the organ fundamentally causing the disease. Moreover, such knowledge should allow for prediction and prevention of other related-organ disorders when a patient suffers from a particular organ disorder. Indeed, *H. pylori* infection not only affects the stomach, but is associated with other risk factors for CKD: hypertension, metabolic syndrome, DM, cardiovascular disease, changes in lipid profile [11]. In this study, we focused on the relationship between the kidneys and the stomach, in terms of *H. pylori* infection and/or AG.

Previous meta-analyses have reported that association of *H. pylori* infection and CKD and revealed no association between *H. pylori* infection and either non-dialysis [20] or dialysis-dependent patients [21]. On the other hand, a meta-analysis evaluated the prevalence of *H. pylori* infection in patients with CKD and concluded that there is a lower prevalence of *H. pylori* infection in patients with CKD [22]. The reported risk of CKD in patients with *H. pylori* infection is still conflicting. Given that *H. pylori* inhabits the stomach, it is necessary to consider the state of AG when demonstrating the relationship between *H. pylori* infection and CKD. Although it has been that the risk factor for peptic ulcer are *H. pylori* infection [23], the rate of *H. pylori* infection is lower in individuals with peptic ulcer disease and concomitant CKD than in those without CKD [12]. Thus, it has been suggested that gastric disorders, such as peptic ulcer disease, may influence the relationship between CKD and *H. pylori* infection. Indeed, in the present study, the HP (+) AG (−) and HP (+) AG (+) groups demonstrated an inverse association with CKD. If we examine only the presence or absence of *H. pylori* infection and the risk of CKD without considering the presence or absence of AG, the results are inconsistent. Furthermore, in the 50–59-year-old age stratum, the HP (+) AG (−) group had a CKD prevalence approximately double that of the control group. CKD prevalence and *H. pylori* infection are strongly associated with age [24,25]. This study also showed CKD prevalence was greater among 60–69-year-olds than other age stratum. The effect of the *H. pylori* infection without AG may have been accelerated the onset of CKD in the 50–59-year-old age stratum.

**Figure 2:**
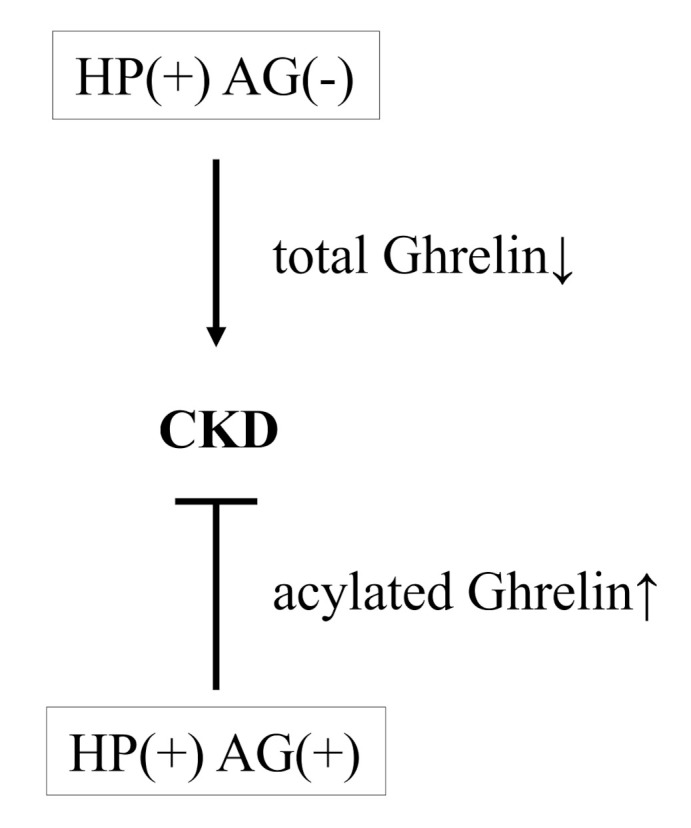
The hypothesis in this study.

These results suggest that the prevalence of HP (+) AG (−) status is higher in CKD and may have relationship with the risk of CKD, an effect that has been speculated by one mechanism: that *H. pylori* infection reduces ghrelin secretion. Ghrelin, a gastrointestinal peptide hormone, is known to perform a plethora of central and peripheral actions in distinct areas, including gut motility, gastric acid secretion, and glucose metabolism [26]. There are two different circulating forms of this peptide: acylated ghrelin, the active form that binds to receptors, and des-acylated ghrelin, the inactive form without affinity for receptors [27,28]. The level of acylated ghrelin is more important than total ghrelin in terms of clarifying the effect of ghrelin. Ghrelin can improve CKD via complex interactions affecting energy homeostasis, appetite, muscle mitochondrial activity, suppression of inflammation, and maintenance of the cardiovascular system [29,30]. *H. pylori* infection is associated with decreased ghrelin production and with a reduction in the number of ghrelin-producing cells [31,32]. In contrast, higher levels of acylated ghrelin have been observed in patients with chronic AG compared with healthy participants [33]. This suggests that there may be a compensatory increase in plasma acylated ghrelin concentration in response to AG, a condition that results in a loss of ghrelin-producing cells and an increase in gastric pH [32]. Therefore, in this study, we speculate that the HP (+) AG (−) and HP (+) AG (+) were observed association with CKD may be due to decreased or increased levels of acylated ghrelin caused by *H. pylori* infection with/without AG. Furthermore, there was no difference in the relationship between CKD and lifestyle factors and medical history between the control and HP (+) AG (−) group. In sum, *H. pylori* infection may induce renal dysfunction via reduction of ghrelin, and further studies should be conducted to confirm this hypothesis (Figure 2 showed the hypothesis in this study.).

The limitations of the present study are mainly related to the study design and lack of measured ghrelin and proteinuria. The present study was cross-sectional; thus, we could not investigate the incidence of CKD in relation to *H. pylori* infection. A prospective study is warranted to assess the relationships between CKD and *H. pylori*. Although the study evaluated medical information obtained via self-administered questionnaires, some anti-diabetes drugs used for other reasons or for pre-diabetes, like metformin and we can’t detect diabetics from non-diabetics. Furthermore, we could not show direct evidence of an interaction between ghrelin levels and *H. pylori* infection and/or AG status. In addition, CKD is generally defined in terms of proteinuria and eGFR; however, we had limited data on proteinuria. Last, we diagnosed *H. pylori* infection via serum antibody detection, whereas the gold standard is gastric-based testing.

## 5. Conclusions

We demonstrated that the prevalence of HP (+) AG (−) status is higher in CKD and may have relationship with the risk of CKD. *H. pylori*, an important pathogenic factor in the stomach, is probably involved in the development of many other diseases. Uncovering the association between gastric and renal conditions could lead to the development of new treatment strategies. In theory, if a patient suffers from *H. pylori* infection, we could predict renal disorders and potentially prevent their development by treating the stomach, for example, by administering *H. pylori* eradication treatment.

## Figures and Tables

**Table 1:** Characteristics of subjects according to HP infection and/or AG status.

Variable	HP (−) AG (−)		HP (+) AG (−)		HP (+) AG (+)		HP (−) AG (+)		
	*n* = 2430		*n* = 552		*n* = 517		*n* = 61		*p*-Value
	Mean (n)	SD (%)	Mean (n)	SD (%)	Mean (n)	SD (%)	Mean (n)	SD (%)	
Sex (male)	(744)	(30.6)	(200)	(36.2)	(160)	(30.9)	(23)	(37.7)	0.053
Age (years)	50.0	10.1	54.4	9.80	57.7	8.86	57.5	9.80	<0.001
BMI (kg/m^2^)	21.9	3.06	22.4	3.24	22.1	3.08	22.2	3.64	0.004
Systolic blood pressure (mmHg)	130	20.3	135	20.8	137	21.6	132	21.0	<0.001
Diastolic blood pressure (mmHg)	77.9	11.8	79.9	12.1	80.3	11.5	77.8	13.1	<0.001
Total cholesterol (mg/dL)	213	37.2	219	37.3	218	36.3	216	34.2	0.002
Triglyceride (mg/dL)	128	102	146	102	129	90.8	128	78.4	0.001
HDL-cholesterol (mg/dL)	72.2	19.4	68.4	18.2	69.6	18.6	71.5	20.1	<0.001
LDL-cholesterol (mg/dL)	120	31.6	126	31.4	126	31.6	122	25.7	<0.001
Glucose (mg/dL)	92.3	20.2	94.3	24.4	95.3	22.2	96.0	25.7	0.009
Hemoglobin (g/dL)	13.6	1.41	13.7	1.40	13.5	1.33	13.6	1.71	0.035
Hemoglobin A1C (%)	5.37	0.42	5.46	0.58	5.48	0.49	5.49	0.55	<0.001
Uric acid (mg/dL)	5.19	2.29	5.30	2.32	5.25	2.50	5.59	3.03	0.456
BUN (mg/dL)	13.9	4.04	14.7	4.16	14.2	4.27	14.4	6.32	<0.001
Creatinine (mg/dL)	0.69	0.15	0.72	0.18	0.67	0.14	0.84	1.31	<0.001
eGFR (mL/min/1.73 m^2^)	79.9	13.9	76.1	14.5	79.0	13.8	78.8	17.0	<0.001
PG I (ng/mL)	49.1	19.8	81.9	44.6	44.0	17.2	25.9	16.6	<0.001
PG II (ng/mL)	9.28	3.75	27.3	16.1	23.1	8.60	13.5	6.87	<0.001
PG I/II ratio	5.44	1.28	3.40	1.36	1.92	0.63	1.90	0.84	<0.001
METs (h/day)	12.4	10.3	13.2	10.6	14.1	10.6	16.6	12.6	<0.001
Current smokers	(269)	(11.1)	(68)	(12.3)	(58)	(11.2)	(10)	(16.4)	0.522
Current drinkers	(1484)	(61.1)	(324)	(58.7)	(281)	(54.4)	(34)	(55.7)	0.034
Hypertension	(794)	(32.7)	(232)	(42.0)	(244)	(47.2)	(22)	(36.1)	<0.001
Diabetes mellitus	(58)	(2.4)	(28)	(5.1)	(29)	(5.6)	(4)	(6.6)	<0.001
Dyslipidemia	(790)	(32.5)	(242)	(43.8)	(229)	(44.3)	(21)	(34.4)	<0.001
Anemia	(248)	(10.2)	(51)	(9.2)	(65)	(12.6)	(8)	(13.1)	0.265
Myocardial infarction and/or stenocardia	(33)	(1.4)	(11)	(2.0)	(8)	(1.5)	(1)	(1.6)	0.738
Stroke	(22)	(0.9)	(4)	(0.7)	(6)	(1.2)	(1)	(1.6)	0.825

HP, Helicobactor pylori; AG, atrophic gastritis; BMI, body mass index; HDL, high density lipoprotein; LDL, low density lipoprotein; BUN, blood urea nitrogen; GFR, glomerular filtration rate; PG, pepsinogen; METs, metabolic equivalents. *p*-values were determined using one-way ANOVA or chi-squared test between status.

**Table 2:** The distribution of HP infection and/or AG status and CKD according to year-grade.

Year	HP (−) AG (−)		HP (+) AG (−)		HP (+) AG (+)		HP (−) AG (+)	
	CKD		CKD		CKD		CKD	
	(−)	(+)	(−)	(+)	(−)	(+)	(−)	(+)
35–49	1262	23	179	5	97	2	13	0
	98.2%	1.8%	97.3%	2.7%	98.0%	2.0%	100.0%	0.0%
50–59	516	44	134	21	125	12	15	2
	92.1%	7.9%	86.5%	13.5%	91.2%	8.8%	88.2%	11.8%
60–69	502	83	173	40	263	18	26	5
	85.8%	14.2%	81.2%	18.8%	93.6%	6.4%	83.9%	16.1%

**Table 3:** Multivariate adjusted associations of CKD with HP infection and/or AG.

	CKD		OR ^†^	95% CI ^†^	*p*-Value	OR ^††^	95% CI ^††^	*p*-Value	OR ^§^	95% CI ^§^	*p*-Value
	(−)	(+)									
HP (−) AG (−)	2280	150	Reference			Reference			Reference		
HP (+) AG (−)	486	66	1.465	1.066–2.012	0.018	1.439	1.046–1.979	0.025	1.443	1.047–1.989	0.025
HP (+) AG (+)	485	32	0.610	0.406–0.917	0.017	0.615	0.408–0.926	0.020	0.608	0.402–0.920	0.019
HP (−) AG (+)	54	7	1.126	0.492–2.573	0.779	1.134	0.492–2.614	0.768	1.076	0.456–2.539	0.867

CKD, chronic kidney disease; HP, Helicobactor pylori; AG, atrophic gastritis; OR, odds ratio; CI, confidence interval. † Adjusted for year, sex. †† Adjusted for year, sex, BMI, METs, drinking and smoking. § Adjusted for year, sex, BMI, METs, drinking and smoking, hypertension, DM, dyslipidemia, stroke, myocardial infarction and/or stenocardia, and anemia.

## Data Availability

The data presented in this study are available on request from the corresponding author.
